# Risk Factors for Weaning Failure in COVID-19 Patients

**DOI:** 10.2478/jccm-2023-0021

**Published:** 2023-07-31

**Authors:** Effrosyni Gerovasileiou, Apostolis-Alkiviadis Menis, George Gavriilidis, Eleni Magira, Prodromos Temperikidis, Sofia Papoti, Nikitas Karavidas, Michael Spanos, Epaminondas Zakynthinos, Demosthenes Makris

**Affiliations:** University Hospital of Larissa, Larissa, Greece; G. Papanikolaou Hospital, Thessaloniki, Greece; Sotiria Regional Chest Disease Hospital of Athens, Athens, Greece; Evangelismos Athens General Hospital, Athens, Greece; Evangelismos Athens General Hospital, Cardiology, Athens, Greece; General University Hospital of Larissa, Larissa, Greece

**Keywords:** COVID-19, weaning, risk factors, shock, COPD, SOFA score, ARDS

## Abstract

**Background:**

Data on risk factors associated with mechanical ventilation (MV) weaning failure among SARS-CoV2 ARDS patients is limited. We aimed to determine clinical characteristics associated with weaning outcome in SARS-CoV2 ARDS patients under MV.

**Objectives:**

To determine potential risk factors for weaning outcome in patients with SARS-CoV2 ARDS.

**Methods:**

A retrospective observational study was conducted in the ICUs of four Greek hospitals via review of the electronic medical record for the period 2020–2021. All consecutive adult patients were screened and were included if they fulfilled the following criteria: a) age equal or above 18 years, b) need for MV for more than 48 hours and c) diagnosis of ARDS due to SARS-CoV2 pneumonia or primary or secondary ARDS of other aetiologies. Patient demographic and clinical characteristics were recorded for the first 28 days following ICU admission. The primary outcome was weaning success defined as spontaneous ventilation for more than 48 hours.

**Results:**

A hundred and fifty eight patients were included; 96 SARS-CoV2 ARDS patients. SOFA score, Chronic Obstructive Pulmonary Disease (COPD) and shock were independently associated with the weaning outcome OR(95% CI), 0.86 (0.73–0.99), 0.27 (0.08–0.89) and 0.30 (0.14–0.61), respectively]. When we analysed data from SARS-CoV2 ARDS patients separately, COPD [0.18 (0.03–0.96)] and shock [0.33(0.12 – 0.86)] were independently associated with the weaning outcome.

**Conclusions:**

The presence of COPD and shock are potential risk factors for adverse weaning outcome in SARS-CoV2 ARDS patients.

## Introduction

World Health Organisation declared a Public Health Emergency of International Concern on January 2020 and characterized the outbreak as a pandemic on March 2020 [1]. Since then, there has been a dramatic global spread of the disease, with the cumulative number of confirmed patients exceeding 230 million, while the death toll has reached nearly 5 million [2]. The disease may vary from an asymptomatic infection to acute respiratory distress syndrome (ARDS) and it can be fatal [3]. About 5% of patients present ARDS and require admission to the intensive care unit (ICU).

COVID-19 patients who present severe respiratory failure require invasive mechanical ventilation (IMV) [4]. The duration of mechanical ventilation (MV) in those patients may be prolonged, ranging from 9 to 13.5 days [5,6]. Prolonged MV duration has been associated with high incidence of complications such as infections, barotrauma and increased mortality [7,8]. It is widely accepted that weaning from MV is critical for patients’ mortality and morbidity and international scientific societies have issued guidelines for weaning from MV and reducing ICU stay duration and related complications [9].

Previous studies have identified several risk factors for prolonged MV and unsuccessful weaning [10,11]. However, it is unclear whether COVID-19 ARDS patients have a similar profile and outcomes compared to ARDS patients, or they comprise a distinct population with different risk factors for unsuccessful weaning. In this study, we thus aimed to determine potential risk factors for weaning outcome in patients with SARS-CoV2 ARDS.

## Patients and Methods

### Study design and population

This is a retrospective multicentre observational cohort study carried out between 2020 – 2021. All consecutive adult patients were screened and were included according to the following criteria: a) age greater or equal to 18 years, b) need for MV for more than 48 hours and c) diagnosis of ARDS due to SARS-CoV2 pneumonia or primary or secondary ARDS of other etiologies [12]. Exclusion criteria were: a) insufficient data from the medical records and b) SARS-CoV2 infection without pulmonary involvement. Data were collected from the medical records of 4 Greek Hospitals: Sotiria Thoracic Diseases Hospital of Athens, Evangelismos University Hospital of Athens, AHEPA University Hospital of Thessaloniki and University Hospital of Larissa. National Healthcare System's databases deidentified data and therefore, no patient consent is required and the study is in Institutional Review Board exempt.

### Outcomes

The primary outcome of the study was weaning success defined as spontaneous ventilation for >48 hours within the first 28 days from ICU admission. Secondary endpoints were 28 day mortality, length of ICU stay and ICU MV-free days at the 28^th^ ICU day.

### Data collection

Data were collected using electronic forms which included specific items for data collected. The following data were collected at ICU admission: age, gender, body mass index, admission category (medical, surgical, trauma), SARS-CoV2 infection, comorbidities {Chronic Obstructive Pulmonary Disease (COPD), cirrhosis, chronic dialysis, diabetes mellitus, chronic heart failure, immunosuppression}, cause of ICU admission, clinical severity at ICU admission {Sequential Organ Failure Assessment (SOFA) and Acute Physiology And Chronic Health Evaluation II (APACHE II)}. The following data were collected during the ICU stay: SOFA score, ΔPaO_2_/FiO_2_, mechanical ventilation settings including positive end-expiratory pressure (PEEP), plateau airway pressure, driving pressure (at ICU Day 0, 7, 12, 28), duration of IMV, tracheostomy, extubation (number of attempts), temperature, leucocyte, C-reactive protein (CRP), fluid balance, sedation (type and duration), presence (and type) of infections, antiviral and antibiotic treatment, vasopressors (type and duration), presence (and type) of shock, use of corticosteroids, duration of ICU stay, and ICU mortality. SARS-CoV2 infection was confirmed by positive polymerase chain reaction (PCR) testing of nasopharyngeal or respiratory secretions samples. Lung infections that did not meet the criteria for ventilation acquired pneumonia (VAP) or SARS-CoV2, were classified as pneumonia and different aetiologies were recorded. VAP was defined according to previously published criteria [13,14]. Definition of immunosuppression included: active (under treatment or in remission for less than 5 years) solid cancer, active haematological malignancy, neutropenia <0.7 G/L for >7 days, solid organ transplant, patient receiving steroids (>10 mg equivalent prednisolone) or any other immunosuppressive drug for >28 days, human immunodeficiency virus (HIV), genetic immune deficiency, allogeneic stem cell transplant or organ transplant. Shock diagnosis and classification in different types of circulatory failure (distributive, hypovolemic, cardiogenic, obstructive) [15,16,17] required documentation in medical records and confirmation by relevant clinical signs in medical charts (i.e. mean blood pressure, urine output, use of vasopressors, microbiology).

### Statistical analysis

Quantitive variables were expressed as mean (interquartile range), and categorical variables were presented as absolute numbers and percentage. Patient characteristics at ICU admission and during ICU stay were described and analysed according to the weaning outcome (success or failure). The Mann-Whitney *U* test, the Fisher's exact test and the x^2^ test were applied to analyse the differences between groups according to the type of data. Risk factors associated with death and their odds ratios (OR) were analysed by the univariable logistic regression model. Multivariate analyses were performed to determine variables associated with weaning success. Baseline characteristics associated significantly with successful weaning in univariate analysis were then entered in the multivariate binary regression analysis model. Tests were all two sided. Differences between groups were considered significant when the p value was less than 0.05. SPSS Statistics 22.0 software (IBM. USA) was used for statistical analysis.

## Results

Overall, 158 patients were included in the study, 96 CoVARDS and 62 ARDS patients; forty-two out of 62 patients had primary ARDS secondary to influenza virus pneumonia (n=21), *Streptococcus pneumoniae* pneumonia (n=15) and lung trauma (n=6); twenty out of 62 had secondary ARDS (pancreatitis, n=5, post abdominal operation, n=15). Mortality at the 28^th^ ICU Day in CoVARDS and ARDS groups were 47.9% and 43.5% respectively. CoVARDS patients were hospitalised for a longer period than ARDS patients (21.82 (18.4–25.2) vs 15.25 (13.4–17.03) days, p=0.002 respectively). SOFA score and corticosteroid dose (equivalent to hydrocortisone dose in mg/Kg/day) were significantly higher in CoVARDS group compared to ARDS group [8.24 (7.70–8.77) vs 7.2(6.67–7.75)], p=0.02 and 1.29 (1.04–1.55) vs 0.38 (0.17–0.58) p=0.0001 respectively.

Overall, there were 79 (50%) patients with shock; 52 (65.8%) patients presented distributive-septic shock which was attributed to VAP (n=9), blood stream infection (BSI), (n=22) or to both VAP and BSI (n=21), (Table 1). In 27 patients the cause of shock remained unclear (absence of microbiological documentation or, of other positive diagnostic test or, more than one cause were recorded i.e. cardiogenic and distributive secondary to pharmacologic effects). In CoVARDS group shock aetiology is presented in Table 2.

**Table 1.** Characteristics of participants according to weaning outcome.

	**Weaning Success (N=61)**	**Weaning Failure (N=97)**	**P Value**
Age, years	62.39 (59.1–65.66)	67.41 (65.22–69.61)	0.02
Men, n (%)	42 (68.9)	61 (62.96)	0.49
APACHE II score Day 0	14.87 (13.46–16.28)	16.46 (15.19–17.72)	0.12
SOFA score Day 0	7.2 (6.67–7.75)	8.24 (7.70–8.77)	0.02
Heart failure, n (%)	4 (6.5)	18 (18.5)	0.03
Diabetes mellitus, n (%)	13 (21.3)	14 (14.4)	0.28
Hypertension, n (%)	31 (50.8)	46 (47.4)	0.74
COPD, n (%)	4 (6.5)	20 (20.6)	0.02
Immunosuppression, n (%)	2 (3.2)	12 (12.3)	0.08
Corticosteroids, mg/Kg/Day^a^	0.95 (0.65–1.26)	0.93 (0.69–1.18)	0.66
PO_2_/FiO_2_, mmHg	142.9 (123.2–162.5)	124.9 (97.89–151.9)	0.25
Mechanical ventilation duration, days	7.6 (6.1–9.07)	12.9 (11.06–14.73)	<0.0001
Vt ml/Kg^b^	5.80 (5.30–6.30)	6.26 (6.02–6.51)	0.058
Pplat, ICU Day 0, cmH_2_O	22.85 (21.52–24.18)	25.28 (23.89–26.66)	0.04
Driving Pressure, ICU Day 0, cm H_2_O	11.1 (10.3–11.9)	13.02 (11.84–14.21)	0.03
PEEP ICU Day 0, cmH_2_O	10.54 (9.65–11.42)	10.21 (9.52–10.90)	0.38
PEEP >12 cmH_2_O (Day 0–7), hours	43.53 (28.02–59.03)	55.11 (41.30–68.92)	0.28
Fluid Balance ICU Days 0–7, lit	3.44 (1.17–5.71)	6.17 (4.2–8.13)	0.04
SARS-CoV-2 pneumonia, n (%)	42 (68.8)	54 (55.7)	0.13
Temperature >38.5 °C, hours	21.34 (10.99–31.70)	28.19 (16.70–39.69)	0.56
VAP, n (%)	29 (47.5)	33 (34)	0.09
VAP^c^	153 (17.31)	191 (21.61)	<0.0001
BSI, n (%)	22 (36)	46 (47.4)	0.18
BSI^d^	116 (13.11)	266 (30.06)	0.001
Shock, n (%)	20 (32.7)	59 (60.8)	0.001
Septic – VAP, n (%)	4 (20)	5 (8.5)	
Septic – BSI, n (%)	6 (30)	16 (27.1)	
Septic – VAP&BSI, n (%)	6 (30)	15 (25.4)	
Unclear^*^, n (%)	3 (15)	24 (40.7)	
ICU antibiotics course, days^e^	18.56 (15.78–21.34)	16.55 (14.25–18.84)	0.13
ICU mortality 28^th^ day, n (%)	6 (9.8)	67 (69)	<0.0001
ICU length of stay, days	18.87 (16.17–21.56)	17.25 (14.89–19.61)	0.12

Values are mean (interquartile range) otherwise is indicated.

a Equivalent of hydrocortisone;

b Vt is based on the ideal weight of 75Kg per patient;

c VAP as VAP events / mean of ICU stay * 100;

d BSI as BSI events/ mean of ICU stay * 100;

e At least one antibiotic;

* Absence of microbiological documentation or, of another positive diagnostic test or, more than one cause were recorded i.e. cardiogenic and distributive secondary to pharmacologic effects;

*ICU* Intensive Care Unit, *COPD* Chronic Obstructive Pulmonary Disease, *VAP* Ventilator associated Pneumonia, *BSI* Bloodstream Infection *Vt* Tidal Volume, *ARDS* Acute Respiratory Distress Syndrome, *SOFA* Sequential Organ Failure Assessment, *APACHE II* Acute Physiology and Chronic Health Disease Classification System I, *Pplat* plateau airway pressure, *PEEP* Positive End-Expiratory Pressure

**Table 2.** Characteristics of SARS-CoV2 ARDS patients according to weaning outcome.

	**Weaning Success (N=42)**	**Weaning Failure (N=54)**	**P Value**
Age, years	61.26 (57.12–65.41)	68.98 (65.89–72.08)	0.003
Men, n (%)	28(66.7)	33(61.1)	0.67
APACHE II score ICU Day 0	14.38 (12.95–15.81)	15.69 (14.34–17.03)	0.15
SOFA score ICU Day 0	7.4 (6.80–8.09)	8.2 (7.56–8.84)	0.16
Heart failure, n (%)	2 (4.7)	7 (12.3)	0.29
Diabetes mellitus, n (%)	9 (21.4)	17 (31.5)	0.35
Hypertension, n (%)	20 (47.6)	27 (50)	0.84
COPD, n (%)	2 (4.8)	11 (20.4)	0.03
Immunosuppression, n (%)	0 (0.0)	6 (11.1)	0.03
Corticosteroids, mg/kg/Day^a^	1.13 (0.77–1.49)	1.43 (1.07–1.80)	0.29
PO_2_/FiO_2_, mmHg	148.9 (123.8–174)	106.1 (75.68–136.5)	0.02
Mechanical ventilation duration, days	7.59 (5.91–9.27)	11.56 (9.17–13.92)	0.006
Vt ml/Kg^b^	5.80 (5.27–6.34)	6.39 (6.18–6.11)	0.13
Pplat, ICU Day 0, cmH_2_O	22.67 (21.23–24.1)	25.37 (23.91–26.88)	0.016
Driving Pressure, ICU Day 0, cm H_2_O	10.92 (10.11–11.72)	13.02 (11.84–14.21)	0.017
PEEP ICU Day 0, cmH_2_O	11.45 (10.39–12.51)	11.94 (11.13–12.76)	0.63
PEEP >12 cmH_2_O (Day 0–7), hours	61.5 (41.83–81.17)	82.17 (62.55–101.7)	0.11
Fluid Balance ICU Days 0–7, lit	2.71 (−0.1–5.51)	3.74 (1.17–5.80)	0.3
Temperature >38.5°C, hours	22.36 (10.88–33.83)	14.56 (4.61–24.50)	0.14
VAP, n (%)	22 (53.6)	15 (27.8)	0.01
VAP^c^	126 (19.35)	110 (16.90)	<0.0001
BSI, n (%)	15 (36.6)	25 (46.3)	0.4
BSI^d^	86 (13.23)	183 (28.15)	0.02
Shock, n (%)	12 (29.2)	39 (72.2)	<0.0001
VAP, n (%)	3 (25)	4 (10.2)	
BSI, n (%)	2 (16.6)	11 (28.2)	
VAP and BSI, n (%)	5 (41.6)	8 (20.5)	
Unclear^*^, n (%)	2 (16.6)	15 (38.4)	
ICU antibiotics course, days^e^	16.5 (13.86–19.14)	13.13 (10.60–15.66)	0.02
ICU mortality 28^th^ day, n (%)	4 (9.7)	42 (77.8)	<0.0001
ICU length of stay, days	17.44 (14.87–20)	13.65 (11.18–16.12)	0.01

Values are mean (interquartile range) otherwise is indicated.

a Equivalent of hydrocortisone;

b Vt is based on the ideal weight of 75Kg per patient;

c VAP as VAP events / mean of ICU stay * 100;

d BSI as BSI events/mean of ICU stay * 100;

e At least one antibiotic;

* Absence of microbiological documentation or, of another positive diagnostic test or, more than one cause were recorded i.e. cardiogenic and distributive secondary to pharmacologic effects;

*ICU* Intensive Care Unit, *COPD* Chronic Obstructive Pulmonary Disease, *VAP* Ventilator associated Pneumonia, *BSI* Bloodstream Infection *Vt* Tidal Volume, *ARDS* Acute Respiratory Distress Syndrome, *SOFA* Sequential Organ Failure Assessment, *APACHE II* Acute Physiology and Chronic Health Disease Classification System I, *Pplat* plateau airway pressure, *PEEP* Positive End-Expiratory Pressure.

### Successful weaning

Overall, 61 out of 158 (38.6%) patients weaned successfully from MV; MV duration was 7.63 (6.20–9.07) days and MV free ICU days were 5.76 (4.45–7.08) days. Table 1 shows patients’ characteristics according to the outcome of weaning. ICU mortality at the 28 days was 9.8% in the weaning success group compared to 69% in the weaning failure group (p < 0.0001). Multivariate binary regression analyses showed that COPD [odds ratio (OR), 0.27; (95% CI 0.08–0.89), p = 0.03], shock during ICU hospitalization [0.30 (0.15 – 0.62), p =0.001] and SOFA score on admission [0.86 (0.73 – 0.99), p =0.048] were independently associated with weaning outcome, in the total population (Table 3).

**Table 3.** Results of binary regression analysis of risk factors associated with weaning success in the total population

**Variables**	**S.E**	**P value**	**O.R**	**Lower 95%C.I**	**Upper 95% C.I**
COPD	0.61	0.03	0.27	0.08	0.89
SOFA score	0.08	0.04	0.86	0.73	0.99
Shock	0.37	0.001	0.3	0.14	0.61

*COPD* Chronic Obstructive Pulmonary Disease, *SOFA* Sequential Organ Failure Assessment; *S.E* Standard Error, *O.R* Odds Ratio, *C.I* Confidence Interval

In CoVARDS group, there were 42 out of 96 (43.7%) SARS-CoV2 ARDS patients who presented weaning success. MV duration was 8.5 (6.9–10.2) days in this population. Table 2 shows their characteristics according to the outcome of weaning. ICU mortality was 9.7% in the weaning success and 77.8% in the weaning failure group (p< 0.0001). BSI incidence was significantly lower in the weaning success group than in the weaning failure group [86 (13.23%) vs 183 (28.15%) respectively, p=0.002]. Multivariate binary regression analysis showed that COPD [0.18 (0.03–0.96), p = 0.04) and shock during ICU hospitalization [0.33 (0.13 – 0.86), p =0.02] in CoVARDS patients were independently and negatively associated with the weaning outcome (Table 4).

**Table 4.** Results of binary regression analyses of risk factors associated with weaning success in SARS-CoV2 ARDS patients and ARDS patients

**Variables**	**S.E**	**P value**	**O.R**	**Lower 95% C.I**	**Upper 95% C.I**
SARS-CoV2 ARDS					
COPD	0.85	0.04	0.18	0.03	0.96
SOFA score	0.11	0.17	0.86	0.69	1.06
Shock	0.49	0.02	0.33	0.12	0.86
ARDS					
COPD	0.86	0.36	0.46	0.08	2.44
SOFA score	0.11	0.07	0.82	0.66	1.01
Shock	0.59	0.71	0.8	0.25	2.55

*COPD* Chronic Obstructive Pulmonary Disease, *SOFA* Sequential Organ Failure Assessment; *S.E* Standard Error, *O.R* Odds Ratio, *C.I* Confidence Interval

## Discussion

The main findings of the present multicentre study were: i) COPD, shock and SOFA score, were independently associated with the weaning outcome in the total ARDS population, whereas ii) COPD and shock were independently associated with the weaning outcome in SARS-CoV2 patients.

In this study, we aimed to identify factors associated with the weaning outcome in SARS-CoV2 ARDS mechanically ventilated patients and possible differentiation to usual non COVID-19 ARDS. We found that COPD and shock were independent risk factors for poor weaning outcome in this population. MV duration is known to be significantly longer in COPD patients compared to patients who do not present this airway disease [18]. According to previously reported data in non SARS-CoV2 patients, the incidence of COPD in patients could significantly affect the weaning outcome [19]. However, data regarding COPD and weaning outcome in SARS-CoV2 mechanically ventilated patients are limited. The association between COPD and weaning from MV is in agreement with a recent study reporting that COPD was associated with adverse outcomes such as longer ICU stay and higher mortality, following SARS-CoV2 infection [20]. However, our results were derived from a larger population, from four different Hospitals over a longer period of time.

COPD patients present impaired mechanical properties of their respiratory system that may have an important impact in the weaning from MV [21]. ICU morbidity and mortality are significantly higher in COPD patients compared to non-COPD patients (60% versus 43% respectively, p = 0.027) [22]. Dynamic hyperinflation and the development of PEEP which are observed in patients with COPD and acute respiratory failure, may contribute significantly to increased respiratory load and may affect adversely the work of breathing and consequently the weaning outcome [21]. Moreover, poor outcomes in COPD patients who present respiratory failure related to SARS-CoV2 may be also associated to increased expression of ACE-2 receptors in small airways [23]. More data are needed to reach firm conclusion and to confirm the aforementioned association between COPD and weaning outcome in SARS-CoV2 infected patients.

In the present study, the incidence of shock was significantly lower among patients who had successful weaning from MV compared to the weaning failure group (32.7% vs 60.8%, p=0.001). Shock is frequently encountered in critically ill ARDS populations [24]. In our study the percentage of patients with shock was 45.1% in ARDS and 53.1% in SARS-CoV2 patients. Other studies reported that SARS-CoV2 ARDS patients are more prone to shock than patients with ARDS of other etiologies [25]. Shock is associated with long MV duration or adverse weaning outcome resulting from vasopressors [26], secondary infections [27] and critical illness neuromyopathy [28]. In this respect, it might be those factors and not shock per se that may be independently associated with the weaning outcome. This cannot be excluded in our study. Similarly, Wei et al [29], and Franklin et al [30] underlined that shock during ICU stay is an independent factor for adverse outcomes i.e. need for prolonged ICU stay and high incidence of 28-day mortality.

In our study, among SARS-CoV2 patients, BSI incidence was significantly higher in the weaning failure group 28.2% compared to weaning success group 13.23% (p=0.02). In 76.5% of the SARS-CoV2 patients who suffered shock the main cause was BSI (27.4%) or BSI and VAP (25.5%). Bacterial infections are present frequently in SARS-CoV2 ARDS patients and septic shock may be a potential complication in this setting [31]. In this respect, our findings point out the necessity of strict implementation of infection control measures in the ICU.

Interestingly, in our study, SOFA score – but not APACHE II score- was found to be an independent factor for the weaning outcome in the general population studied here in. However, this association was not significant in the SARS-CoV2 population. This may reflect a different diagnostic performance of the SOFA and APACHE II scores in ICU outcomes in the presence of SARS-CoV2. SOFA score is a tool which is frequently used to assess the severity of patients’ illness during ICU stay and several studies suggested that it may be more reliable than APACHE II score for the weaning outcome[32,33,34].

Other clinical variables such as fluid balance and heart failure, which have been associated with clinical outcomes in previous studies [35], [36], were associated with weaning in the initial univariate analysis but we found no significant association between those variables and the weaning outcome in the multivariate analysis. We believe that both fluid balance and heart failure can have an adverse impact on weaning from MV. However, our population included also septic patients and the impact of fluid balance in weaning may be more difficult to be depicted in these conditions. A larger population might be necessary to depict such an association.

Weaning success in our study was 38.6% whereas weaning failure was associated with significantly higher mortality. Mortality in the CoVARDS group was 47.9%. Several studies have suggested that mortality in these patients may be high as 78% [37,38]. It is well known that weaning failure and prolonged mechanical ventilation may be associated with adverse outcomes in the general ICU population [39]. Weaning failure results in longer duration of MV and longer ICU stay which both are associated with adverse outcome. Zhao et al., [40] reported also a significant association between the weaning outcome and the 28^th^ day mortality in SARS-CoV2 patients.

In the present study we found no differences indicating differences in disease severity (i.e. APACHE, SOFA scores, PO_2_/FiO_2_, incidence of infections or shock) between CoVARDS and ARDS. However, we found significant differences between CoVARDS and ARDS in baseline characteristics such as, PEEP and Driving Pressure levels, corticosteroids, antibiotics duration and prone position used. Those differences may reflect a different approach of clinicians towards CoVARDS; CoVARDS management is challenging and at least at the beginning of the pandemic it was thought as a different type of ARDS. Nevertheless, these differences in the approach of the syndrome in terms of management have not been identified as independent risk factors for the primary outcome in this study and in this respect we believe that these differences are not a factor of bias in our results.

It should be underlined that several points have to be accounted when interpreting the findings of the present study. First, we acknowledge that the size of our population might be small to depict significant differences between specific subgroups. Second, we aimed to include a population that represents the standard population hospitalised in Greek ICUs. In this respect, we have not excluded patients with specific problems i.e. immunosuppression who may be excluded in other similar studies. On the other hand, some information i.e. COPD diagnosis was based on medical records. One might argue that this could lead in underestimation of the true COPD prevalence in our study. We speculate that undiagnosed COPD cases might refer to mild cases [41] and might have not a significant impact on the weaning outcome. Third, MV variables (Driving Pressure and Pplateau), although they were significantly different between patients with weaning success and failure in the univariate analysis, they were not independently associated with the weaning outcome. Both Driving Pressure and Pplateau values in our study were relatively low and within a narrow range (Tables 2 and 3) and this might have obscured their impact on weaning. We certainly acknowledge that both Driving Pressure and Pplateau have been associated with major clinical outcomes in large studies in the past [42,43].

## Conclusion

In summary, this multicentre observational study demonstrated that the presence of COPD and shock were potential risk factors for adverse weaning outcome in ARDS patients regardless of the presence of SARS-CoV2 and in the subpopulation of SARS-CoV2 ARDS patients as well. Our findings suggest also an independent association between illness severity (as expressed by SOFA score) and weaning outcome in ARDS patients. Further prospective studies in larger populations are warranted to investigate the impact of these risk factors on weaning outcome in SARS-CoV2 ARDS patients.
